# Correction to ‘Tissue tropism in parasitic diseases’

**DOI:** 10.1098/rsob.190124

**Published:** 2019-06-26

**Authors:** Sara Silva Pereira, Sandra Trindade, Mariana De Niz, Luisa M. Figueiredo

*Open Biol.* **9**, 190036. (Published online 15 May 2019). (doi:10.1098/rsob.190036)

In table 1, there are two rows with identical title ‘intra-/extracellular’. This has now been corrected to ‘intra-/extra**cellular**’ in line 4 and ‘intra-/extra**vascular**’ in line 5 as the new table shows.

**Table 1.** Summary of tissue involvement in *Trypanosoma* infections.

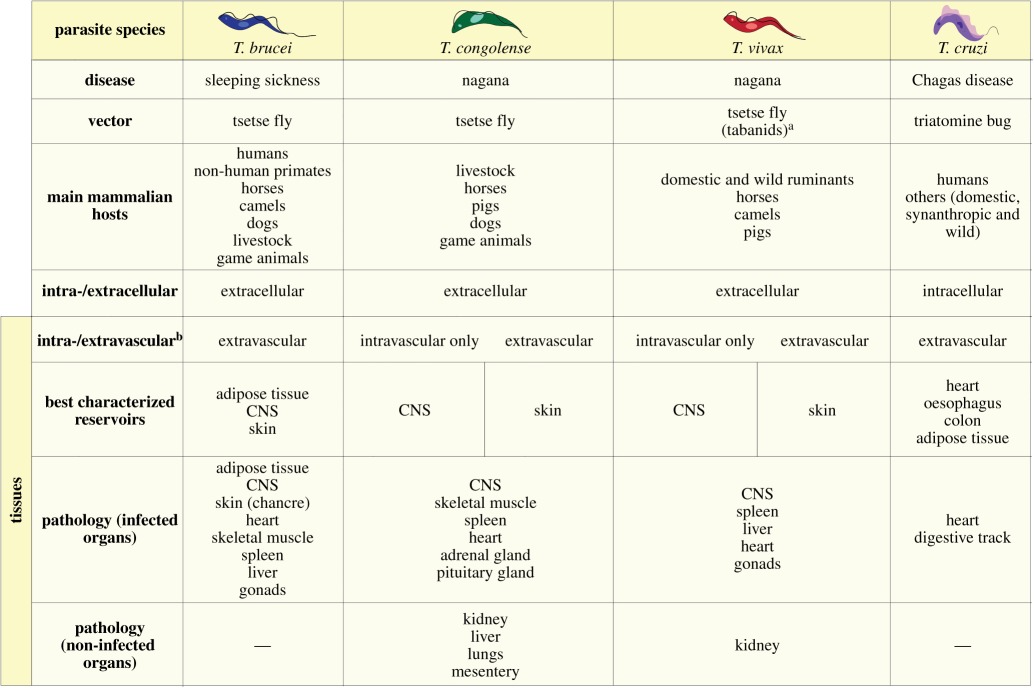

^a^Tabanids are mechanical vectors of *T. vivax*, the parasite cannot differentiate in these insects, but they contribute to transmission in Africa and South America.

^b^Extravascular does not exclude intravascular.

